# Differential Roles of AXIN1 and AXIN2 in Tankyrase Inhibitor-Induced Formation of Degradasomes and β-Catenin Degradation

**DOI:** 10.1371/journal.pone.0170508

**Published:** 2017-01-20

**Authors:** Tor Espen Thorvaldsen, Nina Marie Pedersen, Eva Maria Wenzel, Harald Stenmark

**Affiliations:** 1 Centre for Cancer Biomedicine, Faculty of Medicine, Oslo University Hospital, Oslo, Norway; 2 Department of Molecular Cell Biology, Institute for Cancer Research, Oslo University Hospital, Oslo, Norway; National Cancer Center, JAPAN

## Abstract

Inhibition of the tankyrase enzymes (TNKS1 and TNKS2) has recently been shown to induce highly dynamic assemblies of β-catenin destruction complex components known as degradasomes, which promote degradation of β-catenin and reduced Wnt signaling activity in colorectal cancer cells. AXIN1 and AXIN2/Conductin, the rate-limiting factors for the stability and function of endogenous destruction complexes, are stabilized upon TNKS inhibition due to abrogated degradation of AXIN by the proteasome. Since the role of AXIN1 versus AXIN2 as scaffolding proteins in the Wnt signaling pathway still remains incompletely understood, we sought to elucidate their relative contribution in the formation of degradasomes, as these protein assemblies most likely represent the morphological and functional correlates of endogenous β-catenin destruction complexes. In SW480 colorectal cancer cells treated with the tankyrase inhibitor (TNKSi) G007-LK we found that AXIN1 was not required for degradasome formation. In contrast, the formation of degradasomes as well as their capacity to degrade β-catenin were considerably impaired in G007-LK-treated cells depleted of AXIN2. These findings give novel insights into differential functional roles of AXIN1 versus AXIN2 in the β-catenin destruction complex.

## Introduction

The Wnt signaling pathway orchestrates multiple developmental and adult homeostatic processes, whereas aberrant activation of the pathway underlies numerous human diseases such as cancer [1]. β-catenin, the key mediator of Wnt signaling output [2], is earmarked for proteasomal degradation by the so-called β-catenin destruction complex, which consists of the structural proteins adenomatous polyposis coli (APC) and axis inhibition protein 1 and 2 (AXIN1/2), and the kinases casein kinase 1α (CK1α) and glycogen synthase kinase 3 (GSK3) [3]. This signal-limiting complex is compromised in the majority of colorectal cancers due to mutations in the *APC* gene [4]. Recently, the poly-ADP-ribosyltransferases tankyrase 1 (TNKS1) and tankyrase 2 (TNKS2) were implicated as positive regulators of Wnt signaling by transferring ADP-ribose moieties onto AXIN, the rate-limiting factor for destruction complex stability, thereby marking it for degradation by the ubiquitin-proteasome system [5, 6]. Consequently, tankyrase inhibitors (TNKSi) have emerged as promising new cancer therapeutics that stabilize AXIN and reduce Wnt signaling output [7]. Intriguingly, several studies have reported the formation of distinct cytoplasmic puncta upon treatment with TNKSi [8–10]. These protein assemblies, referred to as degradasomes, contain destruction complex components and most likely represent the morphological and functional correlates of endogenous destruction complexes.

In the current study, we have compared the role of AXIN1 with AXIN2/Conductin in the formation of degradasomes and degradation of β-catenin induced by the TNKSi G007-LK in colorectal cancer cells (SW480), as their relative contribution in the Wnt signaling pathway still remains incompletely understood. Surprisingly, we find that AXIN1 is neither required for G007-LK-induced degradasome formation nor for G007-LK-induced degradation of β-catenin, despite its significant upregulation after prolonged TNKS inhibition (24 h). In contrast, the formation and function of degradasomes were considerably impaired in G007-LK-treated SW480 cells depleted of AXIN2. In addition, new synthesis of AXIN2 was required for degradasome formation upon G007-LK incubation. Taken together, our results imply that AXIN2 is more important than AXIN1 in both initiation of degradasomes and turn-over of β-catenin upon TNKS inhibition.

## Materials and Methods

### Antibodies, plasmids and chemicals

The following reagents were used: rabbit anti-AXIN1 (C95H11), rabbit anti-AXIN2 (76G6), (Cell Signaling Technology); mouse anti-β-catenin (BD Transduction Laboratories); mouse anti-active β-catenin (clone 8E7) (Millipore); mouse anti-β-Actin (Sigma-Aldrich); Hoechst (Invitrogen); G007-LK [11] (gift from Stefan Krauss and Jo Waaler); MG132 (Calbiochem); Cycloheximide (Sigma-Aldrich); Dimethyl sulphoxide (Sigma Aldrich); secondary antibodies for Western blot analysis (IRDye, Li-Cor Biosciences); secondary antibodies for immunofluorescence stainings (Jacksons ImmunoResearch Laboratories or Molecular Probes).

### siRNA transfections

siRNA oligonucleotides were from GE Healthcare Dharmacon. All siRNA transfections were performed using RNAiMax (Invitrogen) according to the manufacturer's protocol and 50 nM siRNA oligonucleotide per well. Non-targeting siRNA used as negative control was from Dharmacon (D-001810-01). For siRNA-mediated depletion of AXIN1 and AXIN2, the following targeting sequences were used: AXIN1: 5’-GGTGTTGGCATTAAAGGTG-3’ [12]; AXIN2: 5’-GAGATGGCATCAAGAAGCA-3’ [13].

### Cell-based assays

The SW480 cell line was purchased from American Type Culture Collection (ATCC). Upon receipt, cells were frozen and individual aliquots were taken into cell culture, typically for analysis within 15 passages. Cells were grown in RPMI medium supplemented with 10% fetal bovine serum and 1% penicillin/streptomycin. Testing for mycoplasma contamination was performed every sixth week. For inhibition of TNKS activity, cells were treated with 0.5 μM G007-LK for indicated time points. Dimethyl sulphoxide (DMSO) was used as a control. For inhibition of proteasomal activity, cells were treated with 10 μM MG132 for 6 h, either alone or in combination with G007-LK. mRNA translation was inhibited by adding 25 μg/ml cycloheximide (CHX) for up to 6 h, either alone or in combination with G007-LK. The SW480 cell line stably expressing GFP-TNKS1 has been described previously [10].

### Western blot analysis

Cells were rinsed in PBS and lysed in Laemmli lysis buffer (65.8 mM Tris-HCl, pH 6.8, 2.1% SDS, 26.3% (w/v) glycerol, 0.01% bromophenol blue, Dithiothreitol (DTT)). Equal amounts of whole cell lysate were separated by SDS polyacrylamide gel electrophoresis (Bio-Rad Laboratories) and blotted onto PVDF membranes (Millipore). Immunodetection was performed with IRDye-conjugated secondary antibodies (LI-COR Biosciences). The Odyssey^®^ Imager system (LI-COR Biosciences) was used to scan all blots. Protein bands were quantified using the Odyssey software.

### Time-lapse live-cell imaging

SW480 cells stably expressing GFP-TNKS1 were treated with G007-LK or a combination of G007-LK and MG132 and then imaged on a Deltavision live cell microscope (Applied Precision, GE Healthcare). To cover the whole cellular volume, stacks were acquired. One stack was acquired before addition of the inhibitors (timepoint "0"), afterwards images were acquired every 10 minutes for 6 h. After deconvolution, sum intensity projections were made. Image analysis was done in ImageJ/Fiji [14] to count the number of GFP-TNKS1 puncta.

### ScanR high-throughput microscopy

Cells were grown on coverslips and fixed in paraformaldehyde. Images were automatically taken using the Olympus ScanR system with an UPLSAPO 40×/0.95 objective. All images were taken with the same settings and below pixel saturation. The Olympus ScanR analysis program was used to measure the average number of G007-LK-induced GFP-TNKS1 puncta. Typically between 2000 and 10000 cells were analyzed per condition in each experiment.

### Statistics

Student`s t-test was done on ScanR and Western blot quantifications to examine statistical significance.

## Results and Discussion

### Proteasome inhibition reduces stabilization of AXIN2 and formation of G007-LK-induced degradasomes

**Fig 1:**
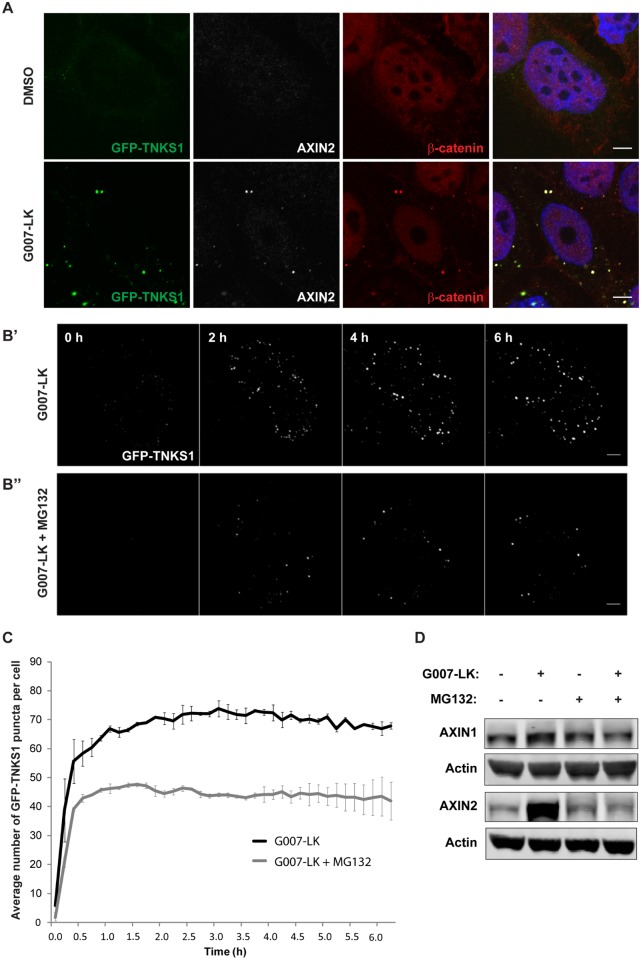
Proteasome inhibition reduces formation of degradasomes during the initial 6 h of TNKSi treatment. (A) Confocal sections through SW480 GFP-TNKS1 cells treated with DMSO (upper panel) or G007-LK (lower panel) for 6 h and immunostained with antibodies against AXIN2, white and β-catenin, red. Hoechst, blue. Scale bar: 5 μm. (B) SW480 GFP-TNKS1 cells were examined by live microscopy after adding G007-LK, either alone (B’) or in combination with MG132 (B”). Images were captured every 10 minutes during a time frame of 6 h. One stack was acquired before addition of the inhibitors (timepoint "0 h"). Still frames of representative cells are shown. Scale bar: 5 μm. (C) Quantification of the number of GFP-TNKS1 puncta per cell. Shown are values +/- SEM of two independent experiments with 24 (G007-LK) and 36 (G007-LK + MG132) cells in total. (D) SW480 cells expressing GFP-TNKS1 were incubated with G007-LK and MG132 for 6 h, either alone or in combination. Cells were then lysed and whole cell lysate was applied for Western blotting. Membranes were incubated with antibodies against AXIN1, AXIN2 and Actin (loading control). One representative blot is shown.

The colorectal cancer cell line SW480 is commonly used as a model for Wnt-dependent cancers due to a mutation in the *APC* tumor suppressor gene [15]. TNKS inhibition antagonizes Wnt signaling in SW480 cells by inducing degradasomes that phosphorylate and earmark β-catenin for proteasomal degradation [8–10]. Degradasomes are rapidly induced upon treatment with TNKSi and contain all components of the endogenous β-catenin destruction complex [10]. Surprisingly, we recently discovered that combining G007-LK with various proteasome inhibitors for 6 h reduced the formation of TNKSi-induced degradasomes [16]. Concurrently, a similar observation was reported upon combination of the TNKSi XAV939 with proteasome inhibitors [17]. To investigate the dynamics of this phenomenon in more detail, we performed live-cell imaging of SW480 cells stably expressing GFP-TNKS1, as TNKS1 localizes to degradasomes and can thus serve as a readout for degradasome formation (Fig 1A, [9, 10]). Time-lapse imaging revealed that degradasomes were rapidly induced (<0.5 h) in cells treated with G007-LK (Fig 1B’ and 1C). Furthermore, we observed a significant reduction (~40%) in degradasome formation when combining G007-LK and MG132 for 6 h compared with G007-LK treatment alone (Fig 1B” and 1C), which is in accordance with previous results from high-throughput microscopy analysis [16]. Interestingly, quantitative image analysis demonstrated that, during the first 30 min of incubation, combined treatment with G007-LK and MG132 increased the number of degradasomes almost to the same extent as treatment with G007-LK alone (Fig 1C). However, in contrast to G007-LK treatment alone, the number of degradasomes was not further increased over the last 5.5 h when combining G007-LK and MG132. Thus, live-cell imaging confirmed the inhibitory role of proteasome inhibitors on G007-LK-induced degradasome formation and added further insight into the rapid-onset kinetics of this process.

AXIN protein levels are regarded as the rate limiting step for the stability and function of endogenous β-catenin destruction complexes [18]. The formation of degradasomes also appears to be AXIN-dependent, although recent studies indicate a role for additional complex components in this process (e.g. TNKS and APC2) [10, 17, 19]. However, the role of AXIN1 versus AXIN2 in TNKSi-induced degradasome formation remains elusive. Although they share the key sequence elements [20, 21], the transcriptional regulation of AXIN1 and AXIN2 differs significantly: in contrast to AXIN1, which is constitutively expressed at very low levels, transcription of AXIN2 is regulated by Wnt signaling activity [22]. To address their relative contribution in the initial formation of degradasomes, we explored the protein levels of AXIN1 and AXIN2 after 6 h G007-LK treatment, either alone or in combination with MG132 (Fig 1D). In line with previous results [10, 16], Western blot analysis revealed a substantial G007-LK-mediated increase in AXIN2 protein levels. However, the protein levels of AXIN1 did not change significantly within the first 6 h of TNKS inhibition. Strikingly, the G007-LK-induced increase in AXIN2 levels was counteracted in the presence of MG132, while AXIN1 protein levels were not affected (Fig 1D, [16]).

Taken together, our findings indicate that formation of degradasomes during the initial 6 h of TNKSi treatment requires a stabilization of AXIN2 protein levels. We further propose that the immediate formation of degradasomes (<30 min) observed in live-cell imaging upon combined G007-LK and MG132 treatment is due to a temporary stabilization of AXIN2 protein levels when inhibiting the proteasome and/or a delay in the MG132-mediated repression of *AXIN2* transcription [16]. However, the formation of degradasomes is abrogated as AXIN2 protein levels fail to be stabilized upon incubation of G007-LK in combination with proteasomal inhibition.

### G007-LK-induced degradasome formation requires sustained protein translation

**Fig 2:**
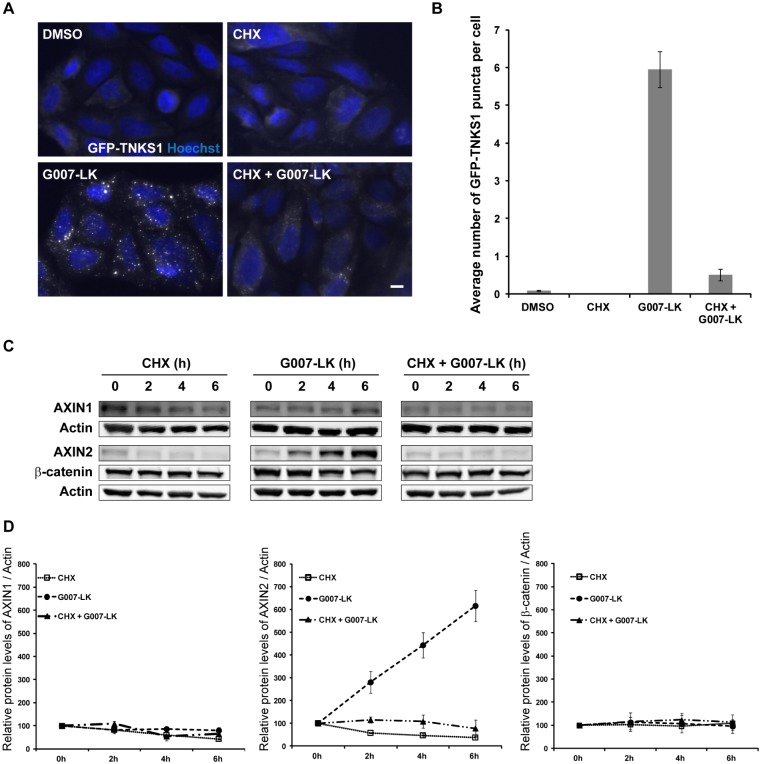
G007-LK-induced formation of degradasomes requires sustained protein synthesis. (A) SW480 cells expressing GFP-TNKS1 were treated with CHX and G007-LK for 6 h, either alone or in combination. Images were taken with the Olympus ScanR microscope. GFP-TNKS1 spots, white; nucleus, blue. Scale bar: 10 μm. (B) The average number of GFP-TNKS1 puncta per cell was quantified using the ScanR analysis software. Shown are values +/- SEM of three independent experiments. At least 2000 cells were analyzed per condition in each experiment. (C) SW480 cells expressing GFP-TNKS1 exposed to the same treatment as in (A) were lysed and whole cell lysate was applied for Western blotting. Membranes were incubated with antibodies against AXIN1, AXIN2, total β-catenin and Actin (loading control). One representative blot is shown. (D) Graphs show quantification of Western blots in (C), from three independent experiments, +/- SEM.

β-catenin, the key mediator of Wnt signaling, initiates transcription of target genes including *AXIN2* by complexing predominantly with the TCF/LEF family of transcription factors [23]. Importantly, transcription of *AXIN2* initiates a negative regulatory feedback loop upon activation of the Wnt pathway [22]. Thus, AXIN2 protein levels are positively regulated via β-catenin-mediated transcription and negatively regulated via TNKS-mediated PARsylation followed by proteasomal degradation. As TNKS inhibition on one hand leads to the degradation of β-catenin and thereby reduced *AXIN2* mRNA transcription, but on the other hand mediates decreased PARsylation and degradation of AXIN2 protein, we sought to further investigate this delicate regulation of AXIN proteins stability upon TNKS inhibition. To elucidate the contribution of AXIN2 new synthesis, we investigated to which extent degradasomes are formed in G007-LK-treated cells after inhibiting mRNA translation with cycloheximide (CHX). SW480 cells expressing GFP-TNKS1 were treated with CHX and G007-LK for 6 h, either alone or in combination, and then investigated by high-throughput image acquisition using the Olympus ScanR microscope (Fig 2A). The number of GFP-TNKS1 puncta was quantified using the ScanR analysis software (Fig 2B). Strikingly, degradasome formation was completely abolished when combining G007-LK with CHX, indicating that G007-LK-induced degradasome formation may require continuous synthesis of AXIN2. Indeed, Western blot analysis revealed that AXIN2 protein levels were not stabilized or increased at any timepoint during 6 h combination treatment (Fig 2C and 2D). In conclusion, our results indicate that the formation of degradasomes requires sustained new synthesis of AXIN2. However, at this point we could not exclude that other, still unknown, initiating factors for degradasome formation may be affected upon inhibition of mRNA translation. Therefore we proceeded with knockdown experiments of AXIN1 and AXIN2.

### AXIN1 is not required for sustained degradasome formation after prolonged TNKSi treatment

**Fig 3:**
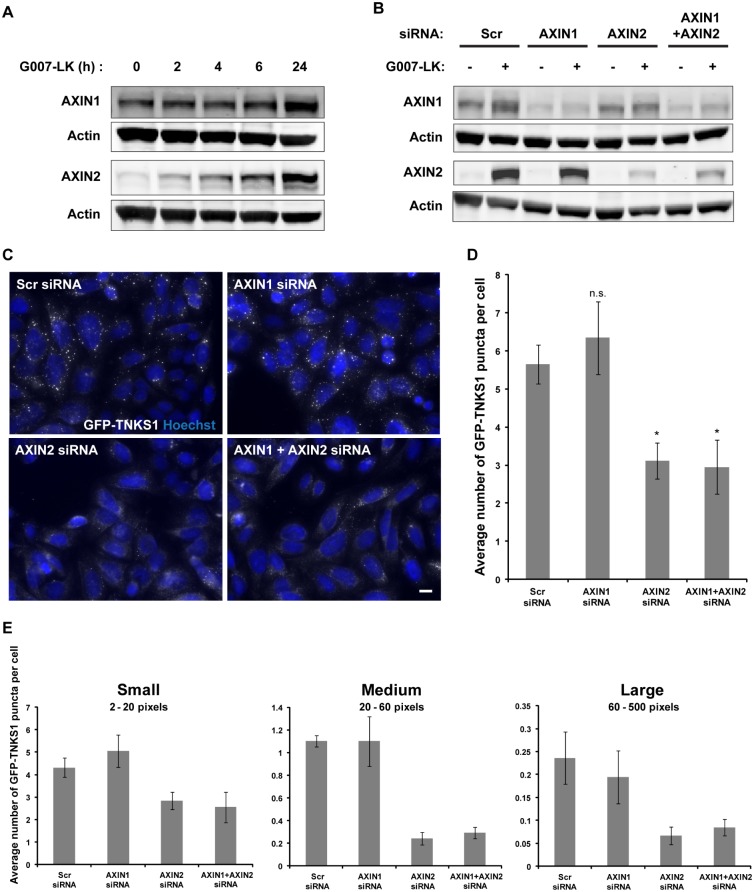
Late-phase upregulation of AXIN1 after prolonged TNKSi treatment is not required for degradasome formation. (A) Western blots of whole cell lysates from SW480 cells treated with G007-LK for 0, 2, 4, 6 and 24 h, respectively. Membranes were incubated with antibodies against AXIN1, AXIN2 and Actin. (B) SW480 cells stably expressing GFP-TNKS1 were treated with G007-LK for 24 h after siRNA-mediated depletion of either AXIN1 or AXIN2, or both proteins for 48 h. Non-targeting siRNA (Scr) was used as a negative control. (C) Images taken with the Olympus ScanR microscope show the formation of GFP-TNKS1 puncta upon G007-LK treatment for 24 h in control (Scr siRNA) cells or cells depleted of AXIN1 and AXIN2, either alone or in combination. Scale bar: 10 μm. (D) The number of GFP-TNKS1 puncta was quantified using the ScanR analysis software. Bars show values +/- SEM from three individual experiments. (E) GFP-TNKS1 puncta from Fig 3D were sorted by size into small (2–20 pixels), medium (20–60 pixels) and large (60–500 pixels) puncta and the average number of puncta per cell in each size category is shown.

The levels of AXIN1 remained essentially unaltered upon 6 h treatment with G007-LK (Fig 1D). However, after prolonged G007-LK treatment (up to 24 h) we observed that both AXIN1 and AXIN2 protein levels were significantly increased (Fig 3A), which is consistent with the effects of the TNKSi XAV939 on AXIN levels after 24 h [9]. To investigate whether this late-phase upregulation of AXIN1 is crucial for degradasome stability and function, SW480 cells stably expressing GFP-TNKS1 were treated with G007-LK for 24 h after short interfering RNA (siRNA)-mediated depletion of either AXIN1 or AXIN2, or both proteins (Fig 3B). Importantly, depletion of AXIN1 did not lead to compensatory up-regulation of AXIN2 or vice versa. For each condition, the capacity of cells to induce degradasomes was investigated with the Olympus ScanR microscope (Fig 3C). The number of GFP-TNKS1 puncta was quantified using the ScanR analysis software and revealed a ~50% reduction in degradasome formation in cells depleted of AXIN2 compared to control cells (Fig 3D). Surprisingly, depleting cells of AXIN1 did not influence the number or size of G007-LK-induced degradasomes and did not significantly further reduce the number of degradasomes in cells depleted for both AXIN1 and AXIN2 (Fig 3D and 3E). We therefore suggest that the 2-3-fold increase in AXIN1 protein level observed after 24 h G007-LK treatment is not required for degradasome formation whereas the 15-20-fold increase in AXIN2 protein level is both necessary and sufficient. Interestingly, AXIN1 is not up-regulated and does not seem to rescue the depletion of AXIN2, despite its higher abundance at steady-state [24] and its moderate increase after 24 h of G007-LK incubation (Fig 3A).

Of note, it was recently demonstrated that a three- to four-fold increase in Drosophila Axin levels is insufficient to inhibit signaling for nearly all Wingless-driven developmental processes, thereby establishing a physiological range within which AXIN levels may fluctuate and still remain compatible with the activation of the pathway following Wnt stimulation in all cells [25]. We suggest that this threshold is not exceeded without the significant increase in AXIN2 levels upon G007-LK treatment. However, additional functional differences between AXIN1 and AXIN2 in degradasomes cannot be excluded.

### AXIN2 depletion counteracts TNKSi-induced reduction in β-catenin protein levels

**Fig 4:**
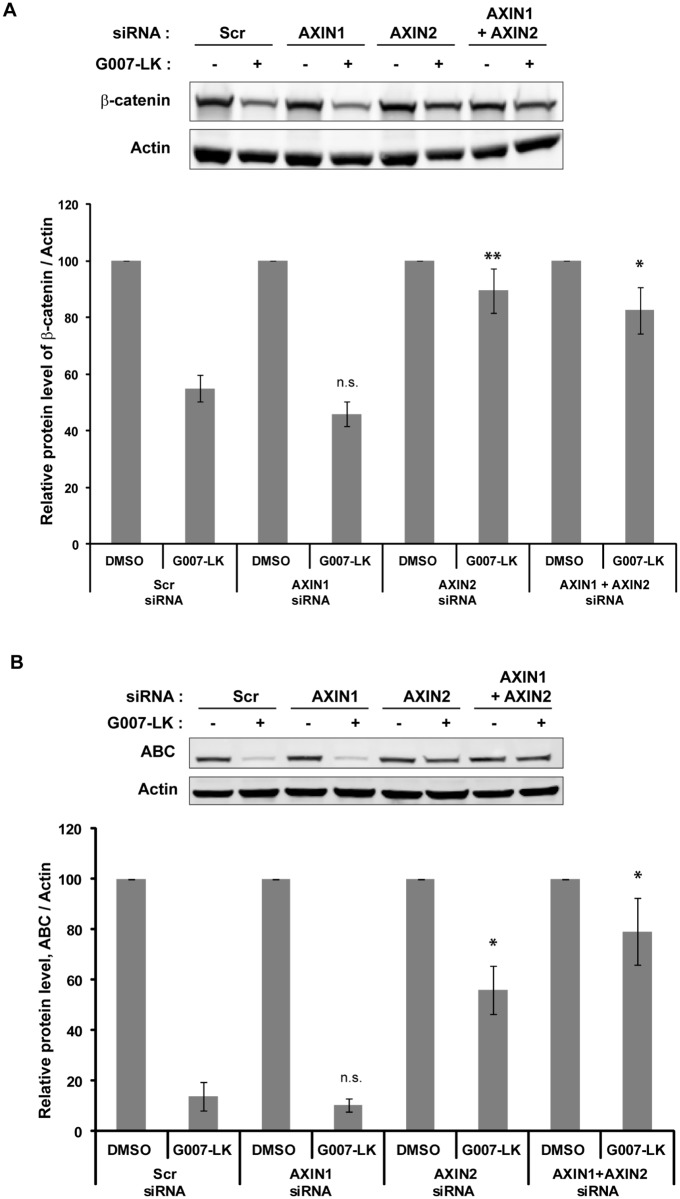
AXIN2 plays a predominant role in degradasome-mediated degradation of β-catenin. (A) SW480 cells stably expressing GFP-TNKS1 were treated with G007-LK for 24 h after depletion of either AXIN1 or AXIN2, or both proteins. The remaining total β-catenin levels were measured by Western blot analysis. Actin was used as a loading control. Quantification of Western blots from three independent experiments, +/- SEM. (B) Lysates from the samples described in (A) were applied for Western blotting. Membranes were incubated with an antibody specifically detecting non-phosphorylated (active) β-catenin. Quantification of Western blots from three independent experiments, +/- SEM.

We recently demonstrated that TNKSi-induced degradasomes in SW480 cells contain phosphorylated β-catenin, ubiquitin and the E3 ubiquitin ligase component β-TrCP, indicating functional degradasomes. Moreover, fluorescence recovery after photobleaching (FRAP) experiments demonstrated that β-catenin-mCherry was rapidly turned over in the G007-LK-induced degradasomes. Taken together, these findings provided a direct mechanistic link between degradasome formation and reduced Wnt signaling in colorectal cancer cells [10]. To further elucidate the individual contribution of the AXIN proteins to the functionality of degradasomes, we investigated whether siRNA-mediated depletion of either AXIN1 or AXIN2 counteracts G007-LK-induced degradation of β-catenin. SW480 cells stably expressing GFP-TNKS1 were treated with G007-LK for 24 h after depletion of either AXIN1 or AXIN2, or both proteins (Fig 3B), and the remaining total and active β-catenin protein levels were measured by Western blot analysis (Fig 4A and 4B). Strikingly, quantification of the protein levels revealed that the G007-LK-mediated reduction in β-catenin protein levels was nearly completely reversed in AXIN2-depleted cells. In contrast, depleting AXIN1 did neither compromise the reduction in β-catenin protein levels induced by tankyrase inhibition nor did it further aggravate the effect of depleting AXIN2 (Fig 4A and 4B). Similar to our results, combined siRNA-mediated depletion of AXIN1 and AXIN2 in SW480 cells reversed the effect of XAV939 on β-catenin degradation and diminished the inhibitory activity of XAV939 in a Super TopFlash reporter assay [6]. However, AXIN1 and AXIN2 were not individually depleted in the study by Huang and colleagues, therefore concealing their individual contributions. Our functional analysis of β-catenin degradation in cells depleted for AXIN1 or AXIN2 indicates a predominant role of AXIN2 in the TNKSi-induced degradation of β-catenin in SW480 cells. As AXIN2 was initially shown to compensate for AXIN1 in a mouse knock-in approach, both proteins were thought to be functionally redundant *in vivo* [26]. However, recent *in vitro* findings from Bernkopf and colleagues report a surprisingly predominant role of AXIN1 in cells stimulated with Wnt ligand [24], which is due to a reduced ability of AXIN2 to polymerize with Dvl2 when compared to AXIN1. This explains how AXIN2 can serve as a negative-feedback regulator of Wnt signaling despite its low abundance. Our study investigates the role of AXIN1 versus AXIN2 in degradasomes by using TNKS inhibition as a means to stimulate β-catenin degradation in APC-mutant cells. Our results indicate a predominant role of AXIN2 compared to AXIN1. We suggest that this can be explained by the severely increased amount of AXIN2 after G007-LK treatment, which induces the polymerization of degradasome components. Polymerization of AXIN1/2, APC and TNKS1/2 are thought to increase avidity for downstream signaling effectors and thus being a prerequisite for efficient β-catenin degradation [27–31]. Thus it seems that AXIN2 may be more important for switching off Wnt signaling, while AXIN1 may predominate in mediating the transition from Wnt off to Wnt on state by being recruited to signalosomes.

In conclusion, we have studied the individual contributions of AXIN1 and AXIN2 in the formation of TNKSi-induced degradasomes and degradation of β-catenin. Taken together our results reveal a surprisingly differential role of AXIN1 and AXIN2. AXIN2 stands out to be the most important player in TNKSi-induced degradasome formation and β-catenin degradation in SW480 colorectal cancer cells. However, the functional role and relative contributions of AXIN1 versus AXIN2 during steady-state turnover of β-catenin in APC wild-type cells remain to be investigated.

## References

[1] Clevers H, Nusse R. Wnt/β-Catenin signaling and disease. Cell. 2012;149(6):1192–205. 10.1016/j.cell.2012.05.012 22682243

[2] Valenta T, Hausmann G, Basler K. The many faces and functions of β‐catenin. EMBO J. 2012;31(12):2714–36. 10.1038/emboj.2012.150 22617422 PMC3380220

[3] Kimelman D, Xu W. beta-catenin destruction complex: insights and questions from a structural perspective. Oncogene. 2006;25(57):7482–91. 10.1038/sj.onc.1210055 17143292

[4] Cancer Genome Atlas Network. Comprehensive molecular characterization of human colon and rectal cancer. Nature. 2012;487(7407):330–7. Epub 2012/07/20. 10.1038/nature11252 22810696 PMC3401966

[5] Wang Z, Tian A, Benchabane H, Tacchelly-Benites O, Yang E, Nojima H, et al The ADP-ribose polymerase Tankyrase regulates adult intestinal stem cell proliferation during homeostasis in Drosophila. Development. 2016;143(10):1710–20. 10.1242/dev.127647 27190037 PMC4874480

[6] Huang S-MA, Mishina YM, Liu S, Cheung A, Stegmeier F, Michaud GA, et al Tankyrase inhibition stabilizes axin and antagonizes Wnt signalling. Nature. 2009;461(7264):614–20. 10.1038/nature08356 19759537

[7] Lehtiö L, Chi N-W, Krauss S. Tankyrases as drug targets. FEBS Journal. 2013;280(15):3576–93. 10.1111/febs.12320 23648170

[8] Waaler J, Machon O, Tumova L, Dinh H, Korinek V, Wilson SR, et al A novel tankyrase inhibitor decreases canonical Wnt signaling in colon carcinoma cells and reduces tumor growth in conditional APC mutant mice. Cancer Res. 2012;72(11):2822–32. 10.1158/0008-5472.CAN-11-3336 22440753

[9] de la Roche M, Ibrahim AEK, Mieszczanek J, Bienz M. LEF1 and B9L shield β-catenin from inactivation by Axin, desensitizing colorectal cancer cells to Tankyrase inhibitors. Cancer Res. 2014;74(5):1495–505. 10.1158/0008-5472.CAN-13-2682 24419084 PMC3947273

[10] Thorvaldsen TE, Pedersen NM, Wenzel EM, Schultz SW, Brech A, Liestøl K, et al Structure, Dynamics, and Functionality of Tankyrase Inhibitor-Induced Degradasomes. Molecular Cancer Research. 2015;13(11):1487–501. 10.1158/1541-7786.MCR-15-0125 26124443

[11] Voronkov A, Holsworth DD, Waaler J, Wilson SR, Ekblad B, Perdreau-Dahl H, et al Structural basis and SAR for G007-LK, a lead stage 1,2,4-Triazole based specific Tankyrase 1/2 inhibitor. J Med Chem. 2013;56(7):3012–23. 10.1021/jm4000566 23473363

[12] Tanneberger K, Pfister AS, Kriz V, Bryja V, Schambony A, Behrens J. Structural and Functional Characterization of the Wnt Inhibitor APC Membrane Recruitment 1 (Amer1). Journal of Biological Chemistry. 2011;286(22):19204–14. 10.1074/jbc.M111.224881 21498506 PMC3103299

[13] Hadjihannas MV, Brückner M, Jerchow B, Birchmeier W, Dietmaier W, Behrens J. Aberrant Wnt/β-catenin signaling can induce chromosomal instability in colon cancer. Proceedings of the National Academy of Sciences. 2006;103(28):10747–52. 10.1073/pnas.0604206103 PMC1502302 16815967

[14] Schindelin J, Arganda-Carreras I, Frise E, Kaynig V, Longair M, Pietzsch T, et al Fiji: an open-source platform for biological-image analysis. Nat Meth. 2012;9(7):676–82. http://www.nature.com/nmeth/journal/v9/n7/abs/nmeth.2019.html#supplementary-information. 10.1038/nmeth.2019 PMC3855844 22743772

[15] Morin PJ, Sparks AB, Korinek V, Barker N, Clevers H, Vogelstein B, et al Activation of β-Catenin-Tcf signaling in colon cancer by mutations in β-Catenin or APC. Science. 1997;275(5307):1787–90. 9065402 10.1126/science.275.5307.1787

[16] Pedersen NM, Thorvaldsen TE, Schultz SW, Wenzel EM, Stenmark H. Formation of Tankyrase Inhibitor-Induced Degradasomes Requires Proteasome Activity. PLoS One. 2016;11(8):e0160507 10.1371/journal.pone.0160507 27482906 PMC4970726

[17] Martino-Echarri E, Brocardo MG, Mills KM, Henderson BR. Tankyrase Inhibitors Stimulate the Ability of Tankyrases to Bind Axin and Drive Assembly of β-Catenin Degradation-Competent Axin Puncta. PLoS ONE. 2016;11(3):e0150484 10.1371/journal.pone.0150484 26930278 PMC4773256

[18] Lee E, Salic A, Krüger R, Heinrich R, Kirschner MW. The roles of APC and Axin derived from experimental and theoretical analysis of the Wnt pathway. PLoS Biol. 2003;1(1):e10 10.1371/journal.pbio.0000010 14551908 PMC212691

[19] Croy HE, Fuller CN, Giannotti J, Robinson P, Foley AVA, Yamulla RJ, et al The PARP enzyme Tankyrase antagonizes activity of the β-catenin destruction complex through ADP-ribosylation of Axin and APC2. Journal of Biological Chemistry. 2016. 10.1074/jbc.M115.705442 PMC4933447 27068743

[20] Behrens J, Jerchow B-A, Würtele M, Grimm J, Asbrand C, Wirtz R, et al Functional interaction of an Axin homolog, Conductin, with β-catenin, APC, and GSK3β. Science. 1998;280(5363):596–9. 9554852 10.1126/science.280.5363.596

[21] Zeng L, Fagotto F, Zhang T, Hsu W, Vasicek TJ, Perry WL Iii, et al The Mouse Fused Locus Encodes Axin, an Inhibitor of the Wnt Signaling Pathway That Regulates Embryonic Axis Formation. Cell. 1997;90(1):181–92. 10.1016/S0092-8674(00)80324-4. 9230313

[22] Leung JY, Kolligs FT, Wu R, Zhai Y, Kuick R, Hanash S, et al Activation of AXIN2 Expression by β-Catenin-T Cell Factor: A FEEDBACK REPRESSOR PATHWAY REGULATING Wnt SIGNALING. Journal of Biological Chemistry. 2002;277(24):21657–65. 10.1074/jbc.M200139200 11940574

[23] MacDonald BT, Tamai K, He X. Wnt/β-Catenin signaling: components, mechanisms, and diseases. Dev Cell. 2009;17(1):9–26. 10.1016/j.devcel.2009.06.016 19619488 PMC2861485

[24] Bernkopf DB, Hadjihannas MV, Behrens J. Negative-feedback regulation of the Wnt pathway by conductin/axin2 involves insensitivity to upstream signalling. Journal of Cell Science. 2015;128(1):33–9. 10.1242/jcs.159145 25380820

[25] Wang Z, Tacchelly-Benites O, Yang E, Thorne CA, Nojima H, Lee E, et al Wnt/Wingless Pathway Activation Is Promoted by a Critical Threshold of Axin Maintained by the Tumor Suppressor APC and the ADP-Ribose Polymerase Tankyrase. Genetics. 2016;203(1):269–81. Epub 2016/03/16. 10.1534/genetics.115.183244 26975665 PMC4858779

[26] Chia IV, Costantini F. Mouse axin and axin2/conductin proteins are functionally equivalent in vivo. Molecular and cellular biology. 2005;25(11):4371–6. 10.1128/MCB.25.11.4371-4376.2005 15899843 PMC1140612

[27] Bienz M. Signalosome assembly by domains undergoing dynamic head-to-tail polymerization. Trends Biochem Sci. 2014;39(10):487–95. 10.1016/j.tibs.2014.08.006 25239056

[28] Fiedler M, Mendoza-Topaz C, Rutherford TJ, Mieszczanek J, Bienz M. Dishevelled interacts with the DIX domain polymerization interface of Axin to interfere with its function in down-regulating beta-catenin. Proceedings of the National Academy of Sciences of the United States of America. 2011;108(5):1937–42. 10.1073/pnas.1017063108 21245303 PMC3033301

[29] Mariotti L, Templeton CM, Ranes M, Paracuellos P, Cronin N, Beuron F, et al Tankyrase Requires SAM Domain-Dependent Polymerization to Support Wnt-beta-Catenin Signaling. Mol Cell. 2016;63(3):498–513. 10.1016/j.molcel.2016.06.019 27494558 PMC4980433

[30] Mendoza-Topaz C, Mieszczanek J, Bienz M. The Adenomatous polyposis coli tumour suppressor is essential for Axin complex assembly and function and opposes Axin's interaction with Dishevelled. Open biology. 2011;1(3):110013 10.1098/rsob.110013 22645652 PMC3352083

[31] Riccio Amanda A, McCauley M, Langelier M-F, Pascal John M. Tankyrase Sterile α Motif Domain Polymerization Is Required for Its Role in Wnt Signaling. Structure. 2016;24(9):1573–81. 10.1016/j.str.2016.06.022. 10.1016/j.str.2016.06.022 27499439 PMC5109827

